# Use of an Alignment-Free Method for the Geographical Discrimination of GTPVs Based on the GPCR Sequences

**DOI:** 10.3390/microorganisms9040855

**Published:** 2021-04-16

**Authors:** Tesfaye Rufael Chibssa, Yang Liu, Melaku Sombo, Jacqueline Kasiiti Lichoti, Janchivdorj Erdenebaatar, Bazartseren Boldbaatar, Reingard Grabherr, Tirumala Bharani K. Settypalli, Francisco J. Berguido, Angelika Loitsch, Delesa Damena, Giovanni Cattoli, Adama Diallo, Charles Euloge Lamien

**Affiliations:** 1 Animal Production and Health Laboratory, Joint FAO/IAEA Division of Nuclear Techniques in Food and Agriculture, Department of Nuclear Sciences and Applications, International Atomic Energy Agency, Friedenstrasse 1, A-2444 Seibersdorf, Austria; chibssasafo@gmail.com (T.R.C.); lydialiu329@hotmail.com (Y.L.); T.B.K.Settypalli@iaea.org (T.B.K.S.); F.Berguido@iaea.org (F.J.B.); G.Cattoli@iaea.org (G.C.); 2 National Animal Health Diagnostic and Investigation Centre (NAHDIC), Sebeta P.O. Box 04, Ethiopia; sombomelaku@yahoo.com (M.S.); delesa_damenaa@yahoo.com (D.D.); 3 Institute of Biotechnology, University of Natural Resources and Life Sciences (BOKU), Muthgasse 18, 1190 Vienna, Austria; reingard.grabherr@boku.ac.at; 4 Directorate of Veterinary Services, Ministry of Agriculture, Livestock and Fisheries, Private Bag, Nairobi 00625, Kenya; kasiiti.orengo@gmail.com; 5 Institute of Veterinary Medicine, Mongolian University of Life Sciences, Zaisan, Ulaanbaatar 17024, Mongolia; erdene64j@gmail.com (J.E.); boldoomglvet@yahoo.com (B.B.); 6 Institute for Veterinary Disease Control, Austrian Agency for Health and Food Safety (AGES), Robert Koch-Gasse 17, 2340 Modling, Austria; angelika.loitsch@ages.at; 7 UMR CIRAD INRA, Animal, Santé, Territoires, Risques et Ecosystèmes (ASTRE), CEDEX 05, 34398 Montpellier, France; adama.diallo@cirad.fr; 8 Laboratoire National d’Elevage et de Recherches Vétérinaires, Institut Sénégalais de Recherches Agricoles (ISRA), Hann, Dakar BP 2057, Senegal

**Keywords:** GTPV, alignment-free algorithm, k-mer, G-protein-coupled chemokine receptor

## Abstract

Goatpox virus (GTPV) belongs to the genus Capripoxvirus, together with sheeppox virus (SPPV) and lumpy skin disease virus (LSDV). GTPV primarily affects sheep, goats and some wild ruminants. Although GTPV is only present in Africa and Asia, the recent spread of LSDV in Europe and Asia shows capripoxviruses could escape their traditional geographical regions to cause severe outbreaks in new areas. Therefore, it is crucial to develop effective source tracing of capripoxvirus infections. Earlier, conventional phylogenetic methods, based on limited samples, identified three different nucleotide sequence profiles in the G-protein-coupled chemokine receptor (GPCR) gene of GTPVs. However, this method did not differentiate GTPV strains by their geographical origins. We have sequenced the GPCR gene of additional GTPVs and analyzed them with publicly available sequences, using conventional alignment-based methods and an alignment-free approach exploiting k-mer frequencies. Using the alignment-free method, we can now classify GTPVs based on their geographical origin: African GTPVs and Asian GTPVs, which further split into Western and Central Asian (WCA) GTPVs and Eastern and Southern Asian (ESA) GTPVs. This approach will help determine the source of introduction in GTPV emergence in disease-free regions and detect the importation of additional strains in disease-endemic areas.

## 1. Introduction

Goatpox virus (GTPV) is a DNA virus belonging to the genus Capripoxvirus within the family Poxviridae, together with sheeppox virus (SPPV) and lumpy skin disease virus (LSDV) [1]. GTPV affects goats, sheep and small wild ruminants [2,3].

Although goatpox is a disease of goats, the causative agent can be GTPV or SPPV. Similarly, both GTPV and SPPV can cause sheeppox and, unfortunately, it is not possible to differentiate infections of small ruminants by GTPV or SPPV either clinically or serologically [4] due to cross-reactivity between capripoxviruses (CaPVs). Hence, molecular methods are increasingly being used to genotype and classify CaPVs and undertake molecular epidemiological studies [2,5,6]. 

Together, GTPV and SPPV cause significant economic losses in small ruminants in endemic regions of Africa and Asia and together they make up one of the major impediments to the improvement of goat and sheep productions in the affected areas [7].

Though GTPV is currently confined to Africa and Asia, recent outbreaks of LSDV and SPPV [7] showed that CaPVs could escape their traditional geographical confinements into new areas and cause severe outbreaks. During an incursion of GTPV into a new geographical area, it would be of great importance to identify the source of introduction to implement proper control measures. The identification of a suitable molecular target for discriminating GTPV strains based on their geographical origins can complement field investigations to determine a potential route of introduction during GTPV outbreaks in disease-free areas. This can also help detect the introduction of new strains in disease-endemic areas. 

Previous studies showed that the G-protein-coupled chemokine receptor (GPCR gene), a host range gene, was suitable to classify CaPVs into one of the three species by real-time PCR [5] and by phylogenetic reconstruction [2,8,9,10,11]. Using the GPCR gene, Le Goff et al. (2009) [2] could distinguish three GTPV sub-clusters comprising isolates from different geographical locations, without being able to define the geographical boundaries clearly. The limited number of samples and the absence of isolates from some geographical locations made it impossible to draw definite conclusions.

The GPCR later became a widespread target for molecular characterization of CaPVs [8,9,10,11]. Thus, a large number of sequences were produced by several research groups and made available in public databases. 

Here, we have sequenced the GPCR genes of 12 GTPVs and analyzed them together with 37 GTPV sequences available in public databases. 

Previous analyses using the GPCR relied on conventional sequence alignments and statistical methods for phylogenetic reconstructions. However, those methods usually exclude gap positions or perform poorly when gaps are present in the sequence alignments [12,13,14]. In many cases, these gaps could represent essential information to enable the classification of viruses. For instance, the GPCR sequence alignments showed the presence of a 21-nucleotide deletion in all SPPVs and several GTPVs, as well as a 12-nucleotide deletion in a few isolates of LSDV [2]. The removal of gaps during analysis can result in the loss of information that is essential for comparison.

To avoid this loss of information, alignment-free methods were proposed, including those using the k-mer frequencies to compute the pairwise distances between sequences [13,14,15].

Here, we have used the k-mer frequencies-based distance calculation and heatmaps to compare the GPCR sequences of GTPVs from various geographical locations and classify them according to their geographical origins. 

## 2. Materials and Methods

### 2.1. Sequencing of New Isolates

Twelve isolates (Table 1) of GTPVs from Ethiopia, Kenya, Ghana and Mongolia were acquired and sequenced for this study. Total DNA was extracted from clinical samples and cell culture supernatants and the GPCR gene was amplified as previously described [8]. All pathological samples and viral isolates were handled within the biosafety level 3 facility of the Institute for Veterinary Disease Control, Austrian Agency for Health and Food Safety, Austria.

The positive PCR products were purified and sequenced at LGC Genomics (Berlin, Germany). The sequence data were assembled using Vector NTI 11.5 software (Invitrogen, Waltham, MA, USA). The generated GPCR sequences have been deposited in GenBank under accession numbers MN161836 to MN161847.

### 2.2. Collection of GTPVs for Alignment-Free Method

The sequences generated for twelve new isolates of this study were analyzed with publicly available GTPVs sequences. Nucleotide sequences and deduced amino acid sequences were aligned using the ClustalW algorithm implemented in MEGA7 [16]. The complete GPCR gene sequences of 37 additional GTPVs from various regions were retrieved from GenBank. For phylogenetic reconstructions, sequences representative of LSDV and SPPV were also retrieved from GenBank and included in the analysis.

Firstly, the GPCR nucleotides sequences were aligned using the ClustalW algorithm (codon option) implemented in MEGA 7. The aligned sequences in FASTA format were converted to nexus format using Seaview. Secondly, the Bayesian phylogenetic inference was performed with BEAST v1.8.4 [17]. The BEAUti module was used to generate BEAST files from the sequence alignments in nexus format. The HKY substitution +G nucleotide substitution was used with a UPGMA starting tree. Then, the Markov Chain Monte Carlo method was run with BEAST, for 10,000,000 generations with a sample taken each 10,000 generations. The TRACER programme was used to inspect the log files and determine the optimum number of burn-in based on the Effective Sample Sizes (ESS > 200). TreeAnnotator was used to generate the Maximum Clade Credibility (MCC) after discarding the 5% burn-in. Finally, the tree was visualized with associated meta-data using the ggtree package in R version 3.5.2 [18].

### 2.3. Distances Calculation and Visualization

Only the sequences that clustered within GTPVs group in the phylogenetic analysis were used in the alignment-free analysis.

The multi-fasta file with the GPCR gene sequences of 49 GTPVs was loaded into the pairwise analysis interface of Heatmapper (http://www.heatmapper.ca/pairwise/, accessed on 14 May 2019). Heatmapper [15] was used to compute k-mer frequencies for each sequence and produce the distance matrix table using an alignment-free algorithm. Briefly, k-mer size of 3 was used to compute the k-mer frequencies for each sequence; then the Euclidian distance measurement method was used to calculate the pairwise distances between each pair of sequences. The distance matrix was visualized as a heatmap with “gplots” package in R version 3.5.2 and the Complete-linkage clustering method was implemented to re-order the sequences. 

Additionally, three alignment-based algorithms were used to generate distance matrices (p-distance, Maximum Composite Likelihood (MCL) and Kimura 2-parameter, all implemented in MEGA software) and produce heatmaps. To examine the influence of the gaps, the positions corresponding to the 21-nucleotide deletion of East and South Asian GTPVs were manually removed and the pattern of distance matrix with alignment-free algorithm was analyzed.

## 3. Results

### 3.1. Sequencing and Phylogenetic Reconstructions

We have successfully sequenced the full GPCR gene for all 12 samples collected from both sheep and goats from Ethiopia, Ghana, Kenya and Mongolia and compared that to GTPVs retrieved from GenBank. 

**Figure 1:**
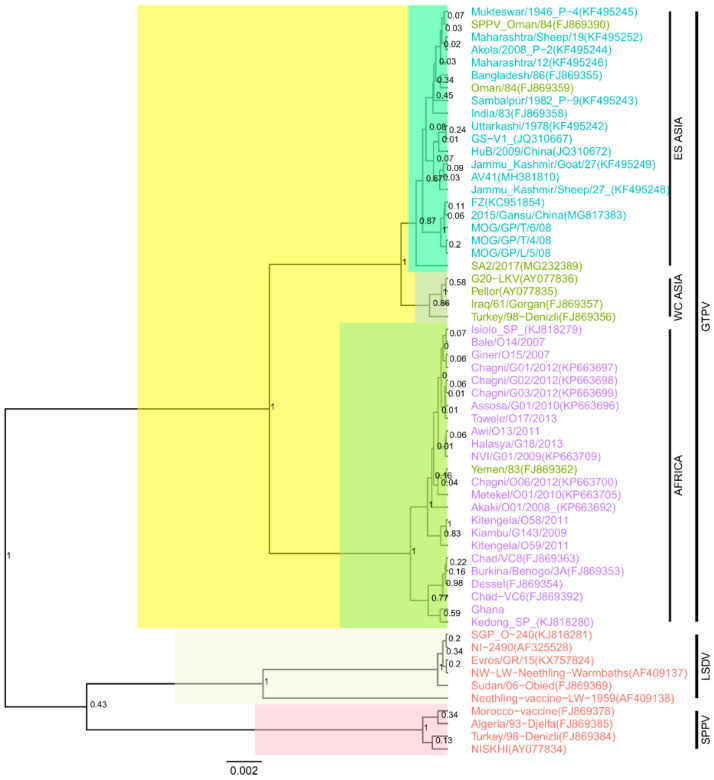
Maximum clade credibility (MCC) tree based on of the complete GPCR complete gene sequences of capripoxviruses. The posterior probabilities are plotted as respective nodes labels. The GTPV sequences are highlighted based their geographical origins (purple for Africa, green for West and Central Asia, blue for East and South Asia). Note GTPV Yemen clustering with African GTPV and GTPV Oman and SPPV Oman, clustering with East and South Asia GTPVs.

The maximum credibility tree of the GPCR sequences showed that all 12 sequences of this study belonged to GTPVs (Figure 1). In the phylogenetic tree, 37 additional sequences retrieved from GenBank clustered with GTPVs. GTPVs formed two distinct groups: one with African isolates and a second one with Asian isolates (Figure 1). The Asian GTPVs produced two sub-clusters: sub-cluster 1 contained GTPVs from Eastern and Southern Asia (ESA) mainly and sub-cluster 2 consisted of isolates of Western and Central Asia (WCA). Among the newly sequenced GTPVs, one isolate from Ghana, five isolates from Ethiopia and three from Kenya, clustered with African GTPVs, while the three GTPVs from Mongolia, clustered with ESA GTPVs (Figure 1). 

Some isolates retrieved from GenBank clustered outside the groups matching their geographical origin. SPPV Oman/84 (FJ869390), GTPV Oman/84 (FJ869359) and GTPV SA2/2017 (MG232389), from Western Asia, clustered with ESA GTPVs, while GTPV Yemen/83(FJ869362) also from Western Asia, clustered with African GTPVs (Figure 1). 

To further determine whether the GPCR gene sequences could help classify GTPVs based on their geographical origins, we selected all 49 GTPVs (37 from GenBank and 12 from this study) for alignment-free sequence comparison.

### 3.2. Distances Calculation and Visualization Using Alignment-Free Methods

**Figure 2:**
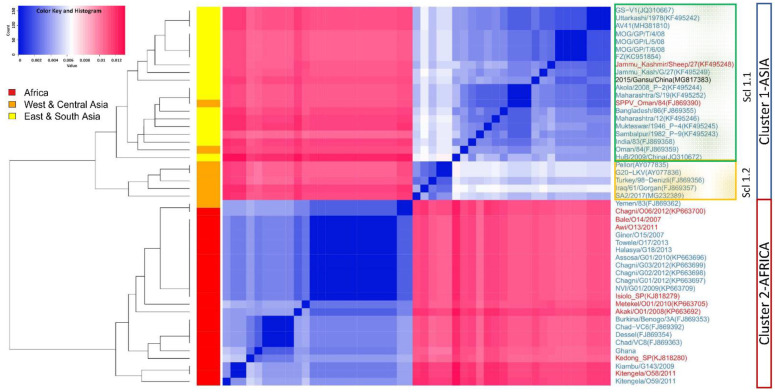
The heatmap for the nucleotides k-mers frequencies variations of 49 GTPVs. The Complete-linkage clustering method was used to re-order the sequences. The vertical side colors indicate the origin of the samples (red for Africa, orange for West and Central Asia, yellow for East and South Asia). The GTPVs recovered from goats are shown in blue, those from sheep in red and those with unknown host in black. The clusters and sub-clusters (Scl) are indicated with boxes.

In the heatmap, based on the Euclidian distances calculated from the k-mer frequencies of each pair of sequences, we observed two main clusters: cluster 1 comprised isolates from Asia and cluster 2 comprised isolates from Africa (Figure 2). Cluster 1 with Asian GTPVs further split into two sub-clusters: sub-cluster 1.1 comprising ESA GTPVs and sub-cluster 1.2 containing WCA GTPVs (Figure 2). In general, all GTPVs clustered according to their geographical origins, except GTPV Yemen/83 (FJ869390) from Western Asia, located within cluster 2 of African GTPVs and GTPV Oman/84(FJ869359) and SPPV Oman/84 (FJ869390), also from Western Asia, located in sub-cluster 1.1 of ESA GTPVs (Figure 2). Contrasting with the result of the phylogenetic reconstruction, GTPV SA2/2017 (MG232389) clustered within sub-cluster 1.1 of WCA GTPVs (Figure 2). 

Heatmaps based on distances calculated from multiple sequence alignments using various substitution models (p-distance, MCL and k2P) produced similar patterns, however with lower resolution. While the two blocks of Asian and African GTPV were apparent, the separation between the two sub-groups of Asian isolates was less prominent and the GTPV SA2/2017 (MG232389) isolate clustered within the ESA GTPVs sub-block 2.1 (Supplement Figure S1). Likewise, after removing the positions containing gaps before calculating the Euclidian distances from the k-mer frequencies, the resulting heatmap, appeared to have a lower resolution, with GTPV SA2/2017 (MG232389) clustering within the ESA GTPVs sub-block 1.2 (Supplement Figure S2).

### 3.3. Amino Acids Profiles of GTPVs’ GPCR

The comparative analysis of the amino acid sequence alignments showed 10 conserved amino acid (aa) differences between African and WCA GTPVs and 17 aa differences (7 aa deletion and 10 aa substitution) between African and ESA GTPVs out of which, nine were common to all Asian GTPVs. The WCA GTPVs differed from ESA GTPVs by seven amino acids, missing in the ESA GTPVs (Supplement Figure S3). In summary, based on the amino acid profile, we can distinguish three different clusters of GTPVs: the African GTPVs, the WCA GTPVs and the ESA GTPVs (Supplement Figure S3). Most of the 49 GTPVs of this study had a GTPV profile that matched the geographical origin except SPPV Oman/84 (FJ869390) and GTPV Oman/84 (FJ869359), which presented the ESA GTPV profile and GTPV Yemen/83 (FJ869390), with the African GTPV profile. Based on the amino acid sequence, GTPV SA2/2017 (MG232389) clustered within the WCA GTPVs sub-group, similar to the result obtained using the heatmap based on the Euclidian distances calculated from the k-mer frequencies.

GTPV SA2/2017 was the only Western Asian isolate sharing an additional amino acid similarity with ESA GTPVs: H was present at position 375 instead of R.

## 4. Discussion

We analyzed the GPCR sequences of GTPVs from various locations, including 12 newly sequenced isolates from Africa and Asia, using multiple sequence alignments and alignment-free methods. 

The analysis of the maximum credibility tree of the GPCR gene sequences confirmed that the newly sequenced isolates belong to GTPVs and enabled the identification of 37 additional GTPVs included for alignment-free analysis. Based on the phylogenetic analysis, the alignment-free sequence comparisons and the information attached to the sequences, we identified two main clusters: the African GTPVs and the Asian GTPVs, which further subdivided into ESA GTPVs and WCA GTPVs. 

In the phylogenetic reconstruction, four isolates, SPPV Oman/84, known to be a GTPV [2], GTPV Oman/84, GTPV Yemen/83 and GTPV SA2/2017, all from Western Asia, clustered outside the groups corresponding to their geographical origin. Contrasting with the phylogenetic analysis, the alignment-free method based on the Euclidian distances calculated from the k-mer frequencies [15], correctly classified GTPV SA2/2017 within the WCA GTPVs, consistently with the results of the comparative analysis of the amino acid profiles of the GTPVs. The failure to correctly classify GTPV SA2/2017 using conventional sequence comparison methods is likely due to the mishandling of the 21-nucleotide deletion observed in ESA GTPVs. Indeed, the heatmaps produced using distance calculated based on conventional methods such as MCL, K2P and p-distance and the one based on k-mer frequencies after removal of the positions with gaps, also failed to classify GTPV SA2/2017 in the correct cluster. Our study further highlights the power of the alignment-free methods in handling useful information such as gaps. Contrasting with global and local alignment algorithms which work base-by-base, frequency-based algorithms can better deal with complexities caused by mismatches, gaps and sequence inversions often found while comparing sequences [13]. 

In a previous study, using thirteen GTPVs GPCR sequences, Le Goff et al. (2009) [2], observed three groups: however, due to the limited number of sequences and the absence of sequences from Eastern Asia and Eastern Africa, they were unable to draw any definite conclusion for the classification of GTPVs based on their geographical origins. 

Though the heatmap based on the alignment-free method provided a better classification of GTPVs according to their geographical origins, this method identified GTPV Yemen/83 as an African GTPV and GTPV Oman/84 and SPPV Oman/84 as ESA GTPVs. 

This misclassification could also suggest that GTPV Yemen/83 is an African GTPV imported to Yemen and GTPV Oman and SPPV Oman are two ESA GTPVs imported to Oman. In the 1980s, when GTPV Yemen/83 was collected, Yemen imported sheep and goats from Eastern Africa, mainly, Somalia, Sudan, Eritrea and Sudan, before the outbreaks [19]. It was suggested that new GTPV strains, imported via Eastern Africa, could have caused these outbreaks [19]. Similarly, Oman imported sheep and goats from Africa, other countries in the Middle East and India, during the same period, with the risk of introducing new GTPVs in the country [19]. The introduction of foreign GTPV strains from Africa to Yemen and/or South Asia to Oman via trade or illegal imports is also consistent with livestock trading routes between African and Asian countries [20]. 

The spread of foot and mouth disease (FMD) in the Middle East, which better illustrates the association between trade and introducing transboundary diseases, was associated with the importation of animals from Asia and Africa leading to epidemics involving new FMD virus strains [20]. The presence of all three GTPV profiles in the Western Asia further supports the hypothesis of GTPVs importation in the region via livestock trade.

These geographical signatures in the GPCR were highly conserved, with no change observed for each region over 50 years. For instance, the WCA GTPVs included in this study were from outbreaks that occurred between 1961 and 2017, the ESA GTPVs, between 1946 and 2009 and the African GTPVs, between 1955 and 2013, implying a great adaptation of these profiles for each region.

The CaPVs’ GPCR gene enabled the discrimination of GTPVs based on their geographical origins. While the GPCR gene encodes for a host factor protein, it remains unclear whether the GPCR profile was driven by the adaptation of GTPV to the affected breeds in each region.

It is also worth noting that some ESA and African GTPVs, including six African GTPVs that were sequenced for this study, were from sheep, showing that these isolates can affect both small ruminant species. In contrast, though only a few GTPVs were available from WCA, we noticed that all of them were exclusively from goats. 

## 5. Conclusions

This study showed that, using alignment-free sequence comparison methods, the GPCR gene sequences enable the classification of GTPVs based on geographical origin. 

The alignment-free approach based on k-mer frequencies enabled a higher resolution as compared to standard approaches using alignment to calculate distance matrix. The result of the alignment-free approach correlates better with the specific features found in the amino acid sequence comparison. 

The approach described here can help support investigations to determine GTPV’s origin of introduction whenever it emerges in a disease-free region and determine the origin of contamination with a new strain in GTPV endemic areas.

## Figures and Tables

**Table 1:** Goatpox viruses used in the comparative analysis.

Isolate	Year of Collection	Species	Country	Region	Accession Number
* Awi/O13/2011	2011	Sheep	Ethiopia	Africa	MN161836
* Bale/O14/2007	2007	Sheep	Ethiopia	Africa	MN161837
* Giner/O15/2007	2007	Goat	Ethiopia	Africa	MN161838
* Towele/O17/2013	2013	Goat	Ethiopia	Africa	MN161839
* Halasya/G18/2013	2013	Goat	Ethiopia	Africa	MN161840
* Kitengela/O58/2011	2011	Sheep	Kenya	Africa	MN161841
* Kitengela/O59/2011	2011	Goat	Kenya	Africa	MN161842
* Kiambu/G143/2009	2009	Goat	Kenya	Africa	MN161843
* Ghana	Unknown	Goat	Ghana	Africa	MN161844
Assosa/G01/2010	2010	Goat	Ethiopia	Africa	KP663696
Chagni/O06/2012	2012	Sheep	Ethiopia	Africa	KP663700
Chagni/G03/2012	2012	Goat	Ethiopia	Africa	KP663699
Chagni/G02/2012	2012	Goat	Ethiopia	Africa	KP663698
Chagni/G01/2012	2012	Goat	Ethiopia	Africa	KP663697
NVI/G01/2009)	2009	Goat	Ethiopia	Africa	KP663709
Yemen/83	1983	Goat	Yemen	West Asia	FJ869362
Metekel/O01/2010	2010	Sheep	Ethiopia	Africa	KP663705
Akaki/O01/2008	2008	Sheep	Ethiopia	Africa	KP663692
Isiolo_SP	1959	Sheep	Kenya	Africa	KJ818279
Chad-VC6	Unknown	Goat	Chad	Africa	FJ869392
Burkina/Benogo/3A	Unknown	Goat	Burkina Faso	Africa	FJ869353
DesseI	Unknown	Goat	Unknown	Africa	FJ869354
Chad/VC8	Unknown	Goat	Chad	Africa	FJ869363
Kedong_SP	1955	Sheep	Kenya	Africa	KJ818280
G20-LKV	2000	Goat	Kazakhstan	Central Asia	AY077836
SA2/2017	2017	Goat	Saudi Arabia	West Asia	MG232389
Iraq/61/Gorgan	1961	Goat	Iraq	West Asia	FJ869357
Pellor	2000	Goat	Kazakhstan	Central Asia	AY077835
Turkey/98-Denizli	1998	Goat	Turkey	West Asia	FJ869356
Maharashtra/12	2008	Goat	India	South Asia	KF495246
Maharashtra/Sheep/19	2010	Sheep	India	South Asia	KF495252
Mukteswar/1946_P-4	1946	Goat	India	South Asia	KF495245
* MOG/GP/L/5/08	2008	Goat	Mongolia	East Asia	MN161845
* MOG/GP/T/4/08	2008	Goat	Mongolia	East Asia	MN161846
* MOG/GP/T/6/08	2008	Goat	Mongolia	East Asia	MN161847
Akola/2008_P-2	2008	Goat	India	South Asia	KF495244
Sambalpur/1982_P-9	1982	Goat	India	South Asia	KF495243
Uttarkashi/1978	1978	Goat	India	South Asia	KF495242
Jammu_Kashmir/Goat/27	2013	Goat	India	South Asia	KF495249
Jammu_Kashmir/Sheep/27	2013	Sheep	India	South Asia	KF495248
GS-V1	2011	Unknown	China	East Asia	JQ310667
India/83	1983	Goat	India	South Asia	FJ869358
SPPV_Oman/84	1984	Sheep	Oman	West Asia	FJ869390
Oman/84	1984	Goat	Oman	West Asia	FJ869359
Bangladesh/86	1986	Goat	Bangladesh	South Asia	FJ869355
FZ	2012	Goat	China	East Asia	KC951854
AV41	2018	Goat	China	East Asia	MH381810
2015/Gansu/China	2015	Unknown	China	East Asia	MG817383
HuB/2009/China	2009	Goat	China	East Asia	JQ310672

* GTPV sequenced for this study.

## Data Availability

The data that support the findings of this study are openly available in NCBI at https://www.ncbi.nlm.nih.gov/nuccore/, accessed on 15 April 2021.

## Supplementary Materials

The following are available online at https://www.mdpi.com/article/10.3390/microorganisms9040855/s1. Figure S1: The heatmap for the nucleotide’s variation of 49 GTPVs based on the MLC distances. The Complete-linkage clustering method was used to re-order the sequences. The vertical side colors indicate the origin of the samples (red for Africa, orange for West and Central Asia, yellow for East and South Asia). Figure S2: The heatmap for the nucleotides k-mers frequencies variations of 49 GTPVs after manual removal of 21 nucleotides from the GPCR genes of WCA and African GTPV. The 21 nucleotides correspond to the gap observed on the multiple sequence alignment in ESA GTPV. The Complete-linkage clustering method was used to re-order the sequences. The vertical side colors indicate the origin of the samples (red for Africa, orange for West and Central Asia, yellow for East and South Asia). Figure S3: Multiple sequence alignment of the complete amino acid sequences of the GPCR of 49 GTPVs from various geographical locations.
