# Supplementary material for: Use of an Alignment-Free Method for the Geographical Discrimination of GTPVs Based on the GPCR Sequences
Source: Microorganisms. 2021 Apr 16;9(4):855. doi: 10.3390/microorganisms9040855 (PMC8073112; doi:10.3390/microorganisms9040855)
Supplement: Supplementary file 1 [file microorganisms-09-00855-s001.zip › Figure S1.pdf]

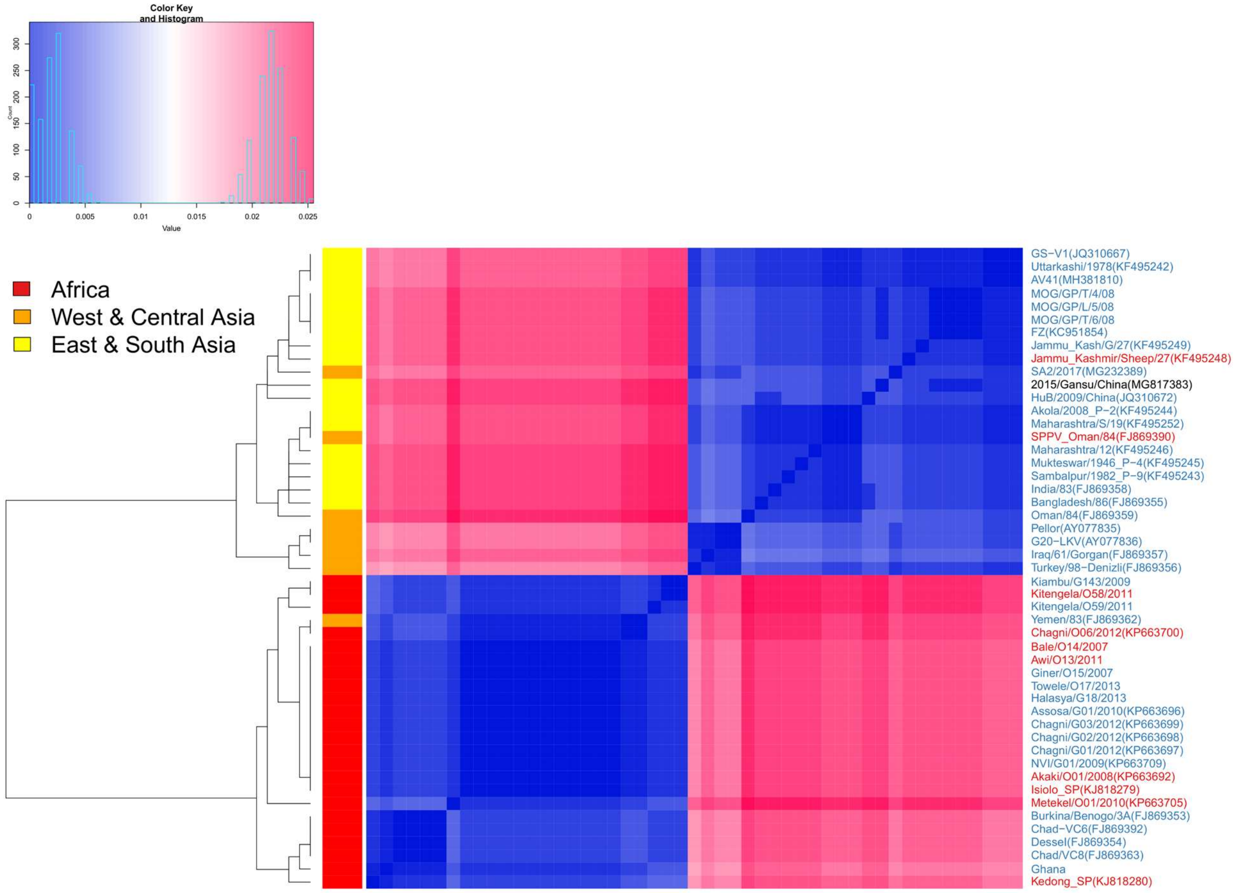

Figure S1. The heatmap for the nucleotide's variation of 49 GTPVs based on the MLC distances. The Complete-linkage clustering method was used to re-order the sequences. The vertical side colors indicate the origin of the samples (red for Africa, orange for West and Central Asia, yellow for East and South Asia).
