# Supplementary material for: Use of an Alignment-Free Method for the Geographical Discrimination of GTPVs Based on the GPCR Sequences
Source: Microorganisms. 2021 Apr 16;9(4):855. doi: 10.3390/microorganisms9040855 (PMC8073112; doi:10.3390/microorganisms9040855)
Supplement: Supplementary file 1 [file microorganisms-09-00855-s001.zip › Figure S2.pdf]

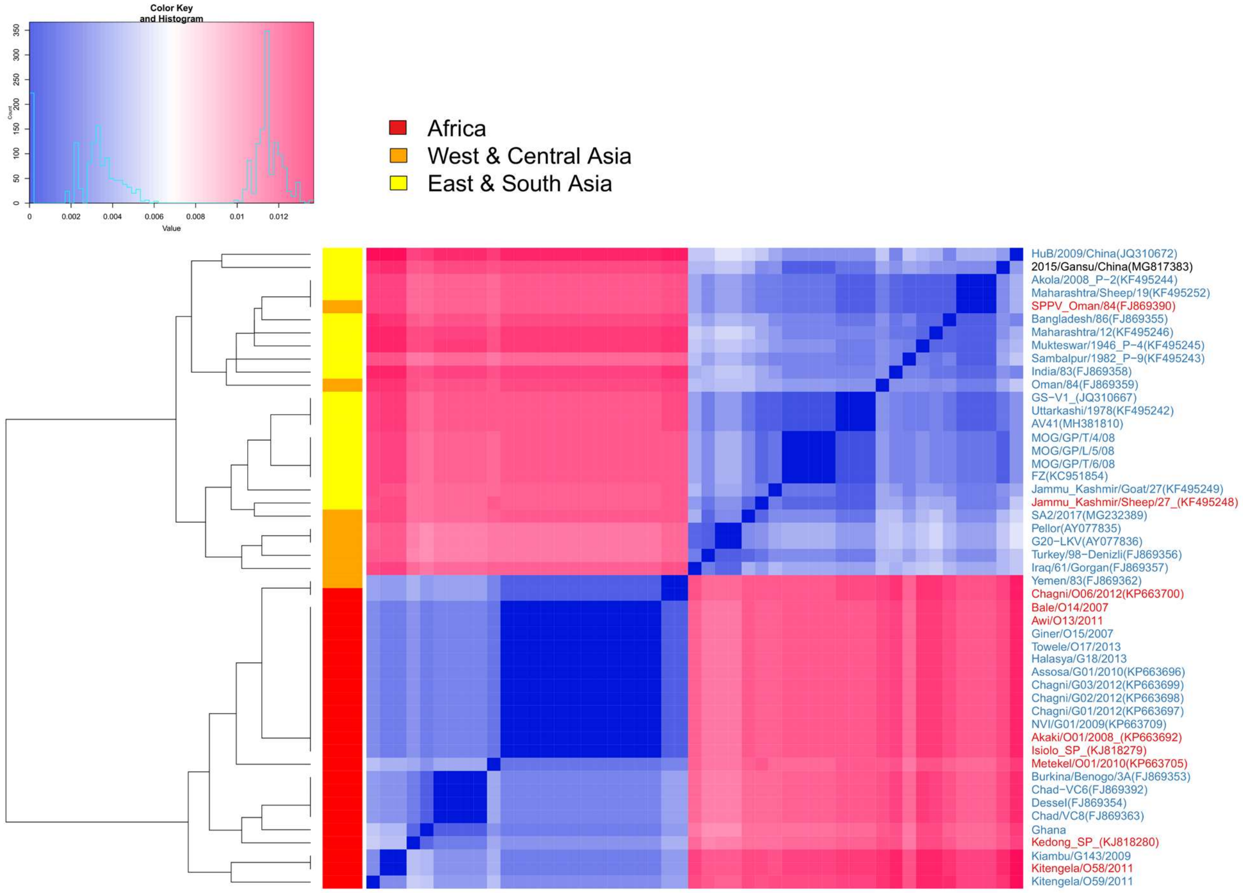

Figure S2. The heatmap for the nucleotides k-mers frequencies variations of 49 GTPVs after manual removal of 21 nucleotides from the GPCR genes of WCA and African GTPV. The 21 nucleotides correspond to the gap observed on the multiple sequence alignment in ESA GTPV. The Complete-linkage clustering method was used to re-order the sequences. The vertical side colors indicate the origin of the samples (red for Africa, orange for West and Central Asia, yellow for East and South Asia).
