# Supplementary material for: Use of an Alignment-Free Method for the Geographical Discrimination of GTPVs Based on the GPCR Sequences
Source: Microorganisms. 2021 Apr 16;9(4):855. doi: 10.3390/microorganisms9040855 (PMC8073112; doi:10.3390/microorganisms9040855)
Supplement: Supplementary file 1 [file microorganisms-09-00855-s001.zip › Figure S3.pdf]

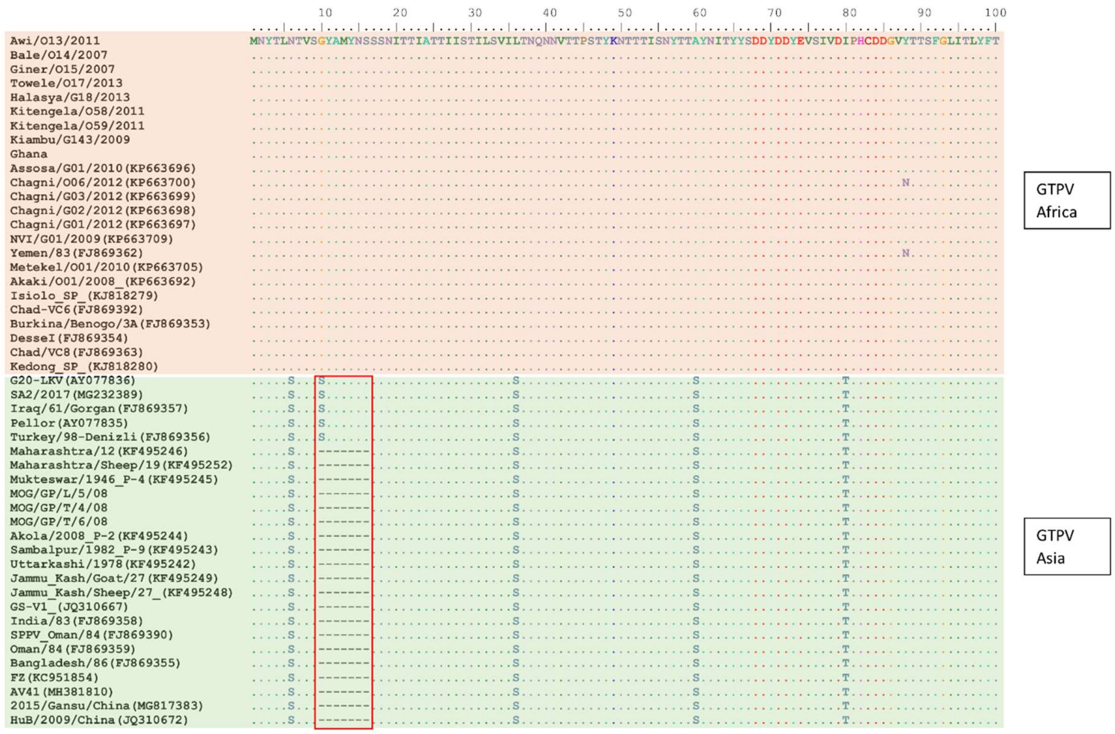

Awi/O13/2011 MNYTLNTVSGYAMYNSSSNITTIATTIISTILSVILTNQNNVTTPSTYKNTTTISNYTTAYNITYYSDDYDDYEVSIVDIPHCDDGVYTTSFGLITLYFT
Bale/O14/2007
Giner/O15/2007
Towele/O17/2013
Halasya/G18/2013
Kitengela/O58/2011
Kitengela/O59/2011
Kiambu/G143/2009
Ghana
Assosa/G01/2010(KP663696)
Chagni/O06/2012(KP663700)
Chagni/G03/2012(KP663699)
Chagni/G02/2012(KP663698)
Chagni/G01/2012(KP663697)
NVI/G01/2009(KP663709)
Yemen/83(FJ869362)
Metekel/O01/2010(KP663705)
Akaki/O01/2008_(KP663692)
Isiolo_SP_(KJ818279)
Chad-VC6(FJ869392)
Burkina/Benogo/3A(FJ869353)
DesseI(FJ869354)
Chad/VC8(FJ869363)
Kedong_SP_(KJ818280)
G20-LKV(AY077836)
SA2/2017(MG232389)
Iraq/61/Gorgan(FJ869357)
Pellor(AY077835)
Turkey/98-Denizli(FJ869356)
Maharashtra/12(KF495246)
Maharashtra/Sheep/19(KF495252)
Mukteswar/1946_P-4(KF495245)
MOG/GP/L/5/08
MOG/GP/T/4/08
MOG/GP/T/6/08
Akola/2008_P-2(KF495244)
Sambalpur/1982_P-9(KF495243)
Uttarkashi/1978(KF495242)
Jammu_Kash/Goat/27(KF495249)
Jammu_Kash/Sheep/27_(KF495248)
GS-V1_(JQ310667)
India/83(FJ869358)
SPPV_Oman/84(FJ869390)
Oman/84(FJ869359)
Bangladesh/86(FJ869355)
FZ(KC951854)
AV41(MH381810)
2015/Gansu/China(MG817383)
HuB/2009/China(JQ310672)
GTPV Africa
GTPV Asia

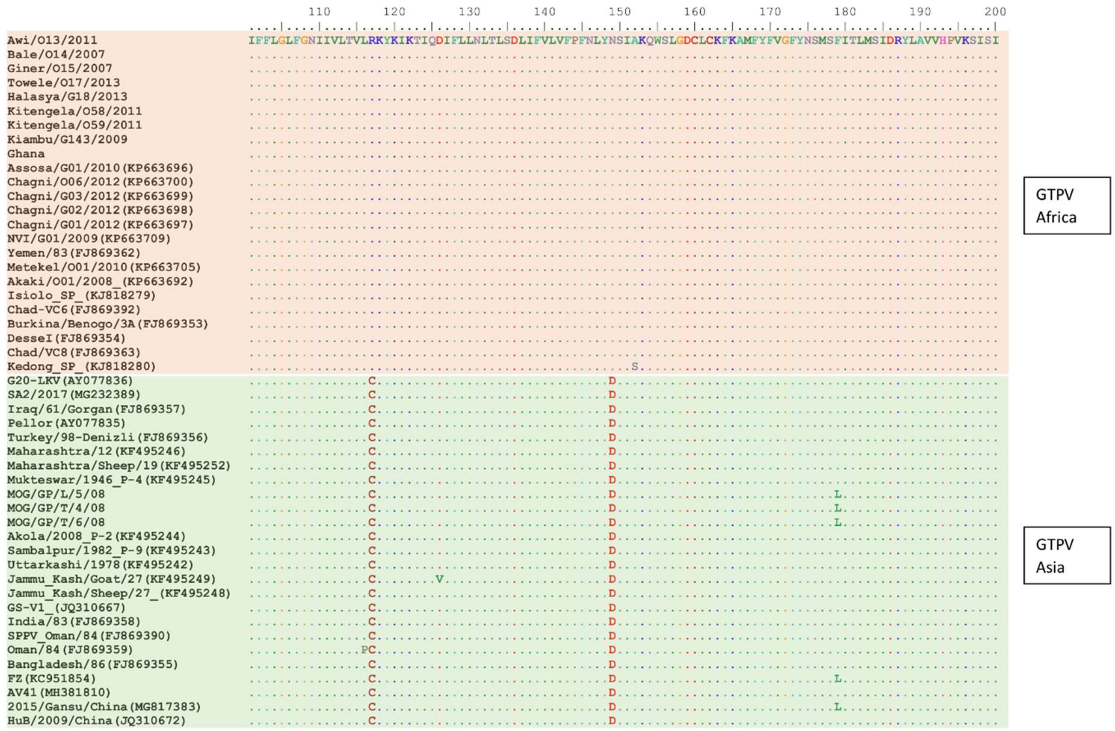

Awi/O13/2011 IFFLGLFGNIIVLTVLRKYKIKTIQDIFLLNLTLSDLIFVLVFPFNLYNSIAKQWSLGDCLCKFKAMFYFVGFYNSMSFITLMSIDRYLAVVHPVKSISI
Bale/O14/2007
Giner/O15/2007
Towele/O17/2013
Halasya/G18/2013
Kitengela/O58/2011
Kitengela/O59/2011
Kiambu/G143/2009
Ghana
Assosa/G01/2010(KP663696)
Chagni/O06/2012(KP663700)
Chagni/G03/2012(KP663699)
Chagni/G02/2012(KP663698)
Chagni/G01/2012(KP663697)
NVI/G01/2009(KP663709)
Yemen/83(FJ869362)
Metekel/O01/2010(KP663705)
Akaki/O01/2008_(KP663692)
Isiolo_SP_(KJ818279)
Chad-VC6(FJ869392)
Burkina/Benogo/3A(FJ869353)
DesseI(FJ869354)
Chad/VC8(FJ869363)
Kedong_SP_(KJ818280)
G20-LKV(AY077836)
SA2/2017(MG232389)
Iraq/61/Gorgan(FJ869357)
Pellor(AY077835)
Turkey/98-Denizli(FJ869356)
Maharashtra/12(KF495246)
Maharashtra/Sheep/19(KF495252)
Mukteswar/1946_P-4(KF495245)
MOG/GP/L/5/08
MOG/GP/T/4/08
MOG/GP/T/6/08
Akola/2008_P-2(KF495244)
Sambalpur/1982_P-9(KF495243)
Uttarkashi/1978(KF495242)
Jammu_Kash/Goat/27(KF495249)
Jammu_Kash/Sheep/27_(KF495248)
GS-V1_(JQ310667)
India/83(FJ869358)
SPPV_Oman/84(FJ869390)
Oman/84(FJ869359)
Bangladesh/86(FJ869355)
FZ(KC951854)
AV41(MH381810)
2015/Gansu/China(MG817383)
HuB/2009/China(JQ310672)
GTPV Africa
GTPV Asia

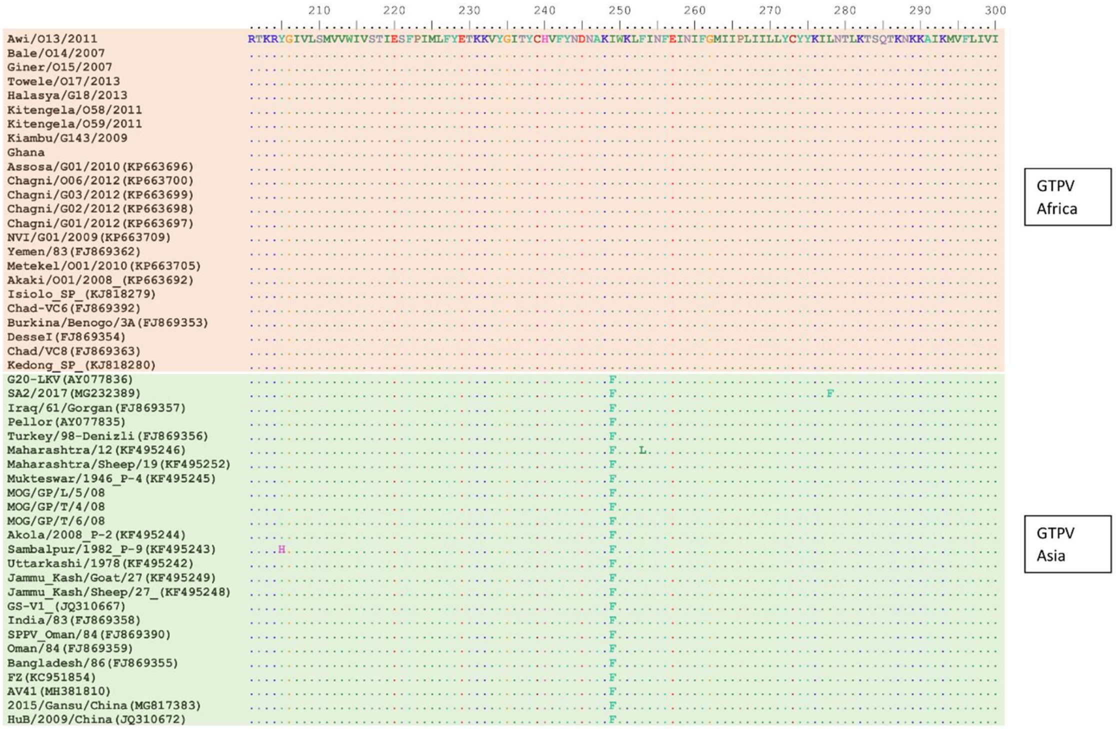

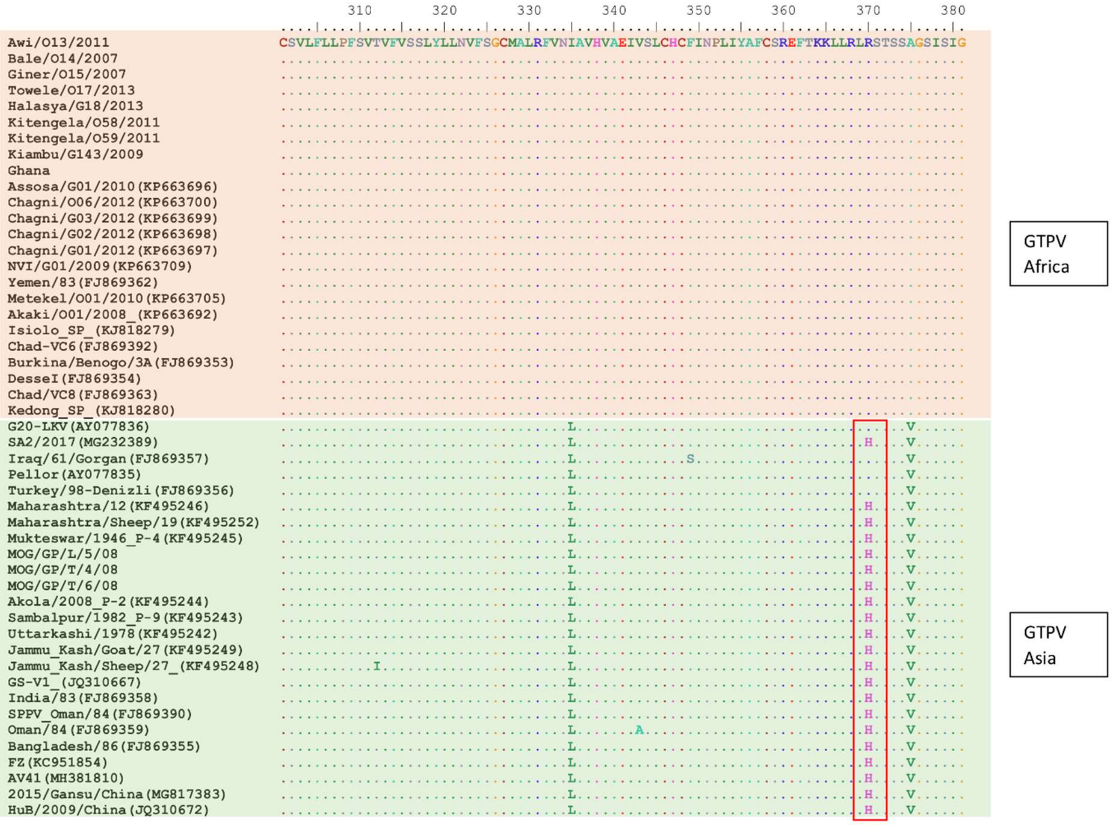

Figure S3. Multiple sequence alignment of the complete amino acid sequences of the GPCR of 49 GTPVs from various geographical locations.
